# Psychogenic Nonepileptic Seizures (PNES) classification: still “time for progress”

**DOI:** 10.1590/0004-282X-ANP-2021-E004

**Published:** 2021-05-07

**Authors:** Luciano De Paola, William Curt LaFrance

**Affiliations:** 1 Universidade Federal do Paraná, Curitiba, PR, Brazil. Universidade Federal do Paraná Universidade Federal do Paraná Curitiba PR Brazil; 2 Brown University; Departments of Psychiatry and Neurology, Providence, Rhode Island, USA. Brown University Brown University Departments of Psychiatry and Neurology Providence Rhode Island USA

Some 20 years ago, in a galvanizing editorial written for the inaugural issue of *Epilepsy and Behavior* journal, John Gates, a pioneer on the field, proclaimed a “time for progress” on nonepileptic seizures^1^. He acknowledged the tremendous progress made during the prior two decades in the field. Nonetheless, he recognized that “much work remained to be done”. Gates’ prophetic words, then, hold true, today. In fact, terminology and classification of nonepileptic seizures encompassed Gates’ initial concerns on that visionary editorial.

Since publication in 2000, roughly 1500 peer-reviewed papers have appeared just on PubMed, specifically on psychogenic nonepileptic seizures (PNES) and its many facets. A recent review identified 15 classification systems for PNES on PubMed listings, from the early 1940s to 2019. The last decade, alone, was punctuated by at least 10 distinct attempts of PNES classification^2^. One of them, the effort by Magaudda *et al*^3^, from three Italian centers, tried to validate a semiologic classification of PNES. The authors essentially selected physical characteristic features of ictal PNES and presented those to both a group of 5 experienced specialists (4 epileptologists and 1 psychiatrist) and to an artificial neural network processing system. They were able to validate 4 distinct semiologic presentations (*i.e*, predominantly hypermotor, akinetic, focal motor or subjective symptoms) as “recognizable and distinguishable”, carrying a high degree of accordance between examiners, in this case using both human and machine learning approaches, reaching 86.7% of cross-validation sets. This work informed the Rosso *et al’s* group to pursue the utility of the classification on patients evaluated at their seizure monitoring unit (SMU) in Buenos Aires, Argentina. =

The conclusions of the Rosso study are presented in this issue of the *Arquivos de Neuropsiquiatria*^*4*^, offering further data on the utility of Magaudda´s proposed semiologic classification, for the general neurologist on daily evaluations. This study presents an opportunity to revisit and update some of the core issues of PNES in this editorial. Not just for those interested on PNES, but those who evaluate patients with all types of ictal events (i.e., epileptic and physiologic nonepileptic), the initiative raises considerations of the validity of new classifications, and of course, the ever challenging issue of cross-cultural aspects in this condition.

The seminal report from the International League Against Epilepsy Nonepileptic Seizures (ILAE) Task Force^5^, defined PNES episodes that, like epileptic seizures, “present as paroxysmal time-limited, alterations in motor, sensory, autonomic, and/or cognitive signs and symptoms, but unlike epilepsy, PNES are not caused by ictal epileptiform activity”. Such diverse clinical presentations almost naturally begs for a “classification” of signs. Inevitably, the expected grounds of a “classification” are based on the easy identification of isolated or clustered semiological signs, hopefully capable of safely differentiating the diagnosis and consistently separating out subgroups with distinct clinical presentations.

This classifying strategy has been tried several times. Similar to the aforementioned works, a recent review by Garg *et al*^6^ analyzed different PNES classification schemes. “Dystonic attacks with primitive gestural activity”, “paucity attacks with preserve responsiveness”, “pseudo-syncope”, “hyperkinetic prolonged attacks”, “axial dystonic prolonged attacks”, “abnormal hypermotor events”, “partial motor events”, “dialeptic type events”, “mixed pattern events”, “pseudo-syncope with or without hyperventilation”, “hyperkinetic prolonged attacks involving limbs and trunk”, “(pure) hypermotor events”, “(pure)non-motor events” and “unclassified events” emerge as some of the potentially identifiable semiologic subgroups. The plethora of terms illustrate the challenge of “classifying” these episodes. Codifying signs can require hours of dedicated analysis of countless video-EEGs (the “gold-standard” tool for diagnosis) taken from the archives of single or combined SMUs. Doing so demands good quality videos and EEG recordings to assure the certainty of PNES diagnosis, homogeneity of patient samples, fair rates of inter-examiner agreement and numbers to achieve statistical validity. The good news, as it seems - and according to Garg *et al* - is that the current, available schemes may classify anywhere from 92.5% up to 100% of PNES. The bad news is that, although promising at first sight, there is ongoing dispute on this topic. While Wadwekar *et al*^7^ feel that there is no need for new classification systems (at least in South India), Duwicquet C *et al*^8^, found only moderate inter-rate reliability of 0.5, for clinical PNES classifications. The authors note the difficult to analyze motor signs and, as expected, concluded that future research is needed, and it would benefit from increased precision of diagnostic criteria.

In reality, not everything is Cartesian when analyzing PNES video samples. There is room for uncertainty regarding: the duration of the event itself, the amplitude of each movement, the validation of one sign over the other whenever they present simultaneously, presence or lack of responsiveness, and the challenging task on interpreting motionless prolonged and discontinuous episodes. Plus, there is a dearth of PNES diagnostic studies in children.

Zhang *et al*^9^ provided information on 88 children diagnosed with PNES and proposed a 5-item classification including “motor symptoms” (rated as the most common on their study), “sensory symptoms”, “unresponsiveness”, “visceral symptoms” and “abnormal behaviors”, the latter being defined as “unusual behaviors similar to psychological symptoms”. The authors identified the additional difficulty of obtaining symptoms from history in the pediatric population. Almost 10 years ago, Szabó *et al*^10^ shared similar concerns on PNES classification in children. This study of 27 children found dialeptic PNES as the most common seizure type, as opposed to more pronounced motor presentations. In addition, almost half of their sample (43%) had emotional - mostly negative - signs during their PNESs.

Variabilities such as these, leave us still in the pursuit of more adequate - hopefully “universal” - PNES diagnostic classifications. Nevertheless, when trying to conceive these studies, future researchers should keep in mind concepts coming from the studies of PNES as a network disorder. A recent review of the functional connectivity literature in PNES^11^ suggested, based on several neuroimaging modalities performed on patients with PNES, that variations in the clinical symptoms may be the result of disruption of various networks. A better understanding of such networks and further neuroimaging studies may provide more definite PNES classifications. A Brazilian group endorses such expectation, as Gallucci-Neto *et al*^12^ described the activation of default model network brain areas and temporoparietal junction as a distinct feature on ictal SPECT studies conducted on 26 PNES patients. In their interpretation, this finding could explain dissociative disorders through an information mismatch between movement prediction input and sensory outcome. Again, the addition of neurobiological input to clinical analysis will eventually lead to the optimal - or best possible - PNES classification.

 Because PNES is a common and disabling disorder, which sometimes has a delay in diagnosis, these features call for continuous state of alert of PNES in the differential diagnosis.

PNES are a global phenomenon across all cultures, not respecting national boundaries, economies or developmental indexes. Despite its broadness, the condition of PNES is still poorly taken by the international academic community, as a unified group. A recent study^13^ found only 12 manuscripts that were truly forged by international consortiums, consisting of 7 studies with patients, 2 surveys and 3 consensus group reports, demonstrating a paucity of cross-cultural collaborations.

As stated by the ILAE Nonepileptic Seizures Task Force^5^, semiologies are described similarly across ethnicities and cultures. Difficulties with diagnosis and management of PNES are likewise similarly distributed, as is the scarcity of resources necessary to the care of these patients. In 2003, Dr. Gates assembled a panel of international faculty during the 25th Epilepsy Congress in Lisbon. It included representatives from United States, Brazil, Taiwan, Lebanon and India to discuss the commonalities and unique cultural expressions of PNES^14^. In the 18 years since this first international conference, and the follow up research in PNES conference that followed in Bethesda in 2005^15^, the concerns raised in Lisbon and in Maryland remain contemporary. Beyond papers such as Rosso et al´s that deal with semiologic classification are opportunities for potentially valuable inter- and intra-continental, multi-national collaborative studies addressing symptomatic therapies, biomarkers, and public health level prevention interventions. These strategic collaborations have the power to prevent years of anguish and iatrogenicity to our patients with PNES. Yes, even presently, there is still “time for progress” in PNES.
